# John Henryism and Cognitive Function and Decline

**DOI:** 10.1093/geroni/igab046.999

**Published:** 2021-12-17

**Authors:** V Eloesa McSorley, Christopher Howard, Bryan James, Raj Shah, Patricia Boyle, Lisa Barnes

**Affiliations:** 1 Rush University Medical Center, Chicago, Illinois, United States; 2 The Utah State Hospital, Provo, Utah, United States; 3 Rush University, Chicago, Illinois, United States; 5 Rush Alzheimer's Disease Center, Chicago, Illinois, United States

## Abstract

Older Black adults in the US have higher prevalence and incidence of dementia and perform lower on cognitive tests than whites. Some of these differences have been attributed to facets of structural racism such as access to and quality of education and fewer socioeconomic resources. Here, we consider whether John Henryism (JH), a measure of self-perceived high-effort coping in the face of chronic environmental and psychosocial stressors, is associated with cognitive function and decline. JH has been associated with adverse cardiovascular health outcomes among African-Americans, especially those with fewer socioeconomic resources. Using data from MARS, we assessed whether JH, measured with an 8-item questionnaire (mean=16.9, sd=4.8, range: 4-27), was associated with level of cognitive function and rate of cognitive decline. We found one standard deviation increase in JH was associated with lower average cognitive function (□=-0.05, 95% CI: -0.09, -0.01). Higher JH was not associated with rate of cognitive decline.

